# Case Report: An Unusual Case of Lemierre’s Syndrome Presenting as Influenza B-Induced Myositis Complicated by Streptococcus intermedius Infection

**DOI:** 10.7759/cureus.64437

**Published:** 2024-07-12

**Authors:** Sophia Perez, Yulia Shtanko, Lorena Del Pilar Bonilla, William Portnoy

**Affiliations:** 1 Medical School, Florida International University, Herbert Wertheim College of Medicine, Miami, USA; 2 Translational Medicine, Florida International University, Herbert Wertheim College of Medicine, Miami, USA; 3 Internal Medicine, Baptist Health South Florida, Miami, USA; 4 Otolaryngology-Head and Neck Surgery, Baptist Health South Florida, Miami, USA

**Keywords:** influenza virus type b, septic thrombophlebitis, streptococcus intermedius bacteremia, lemierre's and lemierre's like syndrome, otolaryngology-head and neck surgery

## Abstract

Lemierre's syndrome (LS) is a rare and severe complication primarily associated with the bacteria *Fusobacterium necrophorum* and characterized by an oropharyngeal infection leading to bacteremia and septic thrombophlebitis. We present a case of an 89-year-old patient with a history of hypertension who initially presented with type B influenza infection and neck pain. She subsequently developed a neck abscess with thrombosis of the internal jugular vein. We believe this to be the first reported case in the literature of LS secondary to *Streptococcus intermedius *presenting after infection with type B influenza. As more atypical LS cases emerge, it is becoming increasingly clear that this condition can manifest in a number of ways. This unique case highlights the importance of considering LS as a differential diagnosis for patients of all ages presenting with neck pain and *Streptococcus intermedius *infection.

## Introduction

Lemierre’s syndrome (LS) is a rare and severe complication of oropharyngeal infections such as bacterial pharyngitis, tonsillitis, mastoiditis, or odontogenic infections. It involves the growth and spread of the bacterial infection to the lateral pharyngeal spaces of the neck, seeding off into the internal jugular vein leading to septic thrombophlebitis and resulting in bacteremia and sepsis [1]. This syndrome is primarily associated with *Fusobacterium necrophorum*, a normal inhabitant of the oral cavity; it is characterized by a range of presentations and complications, reflecting its diverse nature [1-4]. Although rare, recent cases have shown LS development secondary to infection with *Streptococcus intermedius* [5,6]. This β-hemolytic Gram-positive coccus is part of the normal microbiota of the respiratory, gastrointestinal, and genitourinary tracts and is known to cause pyogenic infections of the liver, skin, brain, and heart valves [7,8].

LS typically unfolds in three main phases, starting with an oropharyngeal infection marked by febrile episodes and rigors within four to seven days following the initial illness [1]. In most cases, LS results from oral infections like pharyngitis but can also be secondary to parotitis, otitis media, sinusitis, and mastoiditis [4]. Patients with confirmed Epstein-Barr virus and influenza A infection have also been noted to develop LS [1,9]. It is theorized that the mucosal damage caused by these oropharyngeal infections allows the normal oral flora, such as *Streptococcus intermedius* and *Fusobacterium necrophorum*, to replicate and develop a superinfection which can spread into the lateral pharyngeal spaces and soft tissue of the neck and the bloodstream [1]. The second phase of LS development consists of the infection extending into the parapharyngeal space of the neck and reaching the internal jugular vein. The infection extends into the lateral pharyngeal space, with the posterior compartment housing critical structures such as the carotid artery and internal jugular vein within the carotid sheath [4]. The third and final phase consists of septic emboli [1]. These emboli predominantly affect the lungs, often resulting in the need for mechanical ventilation; however, they can also spread to the liver, joints, kidney, heart, and meninges. 

Diagnosis of LS includes taking a detailed patient history, physical examination findings, diagnostic imaging, and growth of bacteria in blood cultures. Physical examination may reveal signs such as exudative tonsillitis, ulcers, or hyperemia of the pharynx [4]. Computed tomography (CT) neck and chest with contrast can reveal a fully occluded internal jugular vein, retropharyngeal hypodense collection and edema, and potentially metastatic pulmonary septic emboli. Blood cultures should be obtained, though their sensitivity may vary especially when culturing anaerobic organisms [1,4]. While *Fusobacterium necrophorum* is the most common anaerobic organism, other bacteria, such as *Streptococcus *and *Staphylococcus aureus*, can be the root cause; coinfection is also possible [10,11]. 

Antibiotic therapy, which remains the cornerstone of LS treatment, traditionally involves the use of penicillin, clindamycin, metronidazole, and chloramphenicol [4]. A four- to six-week course of carbapenem or piperacillin/tazobactam in combination with metronidazole has been identified as an optimum combination and duration of treatment [10]. However, due to potential variability in antibiotic susceptibility, particularly involving cephalosporins, erythromycin, and penicillin, medication selection should be based on culture and sensitivity results [4]. This emphasizes the importance of tailoring treatment to individual cases. Furthermore, recent studies highlight the need to start antibiotic therapy immediately upon diagnosis [12]. Anticoagulation therapy can also be considered in specific cases, especially when clinical response to antibiotics is inadequate or when thrombophilia and intracranial thrombosis are involved.

While LS most frequently affects young individuals aged 10 to 35 years, cases have been reported across various age groups, highlighting its potential to affect patients of all ages [1-3,13]. Unique presentations of LS have been reported in recent case studies. For instance, an 18-year-old patient was admitted to the intensive care unit due to hypotension, bandemia, and lactic acidosis [3]. Within 12 hours of admission, this patient experienced a rapid clinical decline, developing severe hypoxemia and marked bilateral pulmonary infiltrates on chest X-ray. Mechanical ventilation was required, and the diagnosis of acute respiratory distress syndrome was established. He was found to have LS. Additionally, a 32-year-old man was found to have LS who initially presented with septic shock, fever, abdominal pain, and anuria evolving over five days. Physical examination revealed an obstruction of his suprapubic catheter, which had been inserted six months prior for hypospadias [14].

LS can present in unique capacities with varying sources of infection. Usually, it is more prevalent in younger patients secondary to *Fusobacterium necrophorum*. However, this case demonstrates that it should also be considered in older patients with other anaerobic bacteria infections such as *Streptococcus intermedius*. Additionally, the patient did not initially present with a typical underlying bacterial infection. Rather, she had flulike symptoms, tested positive for influenza type B, and had neck pain. We believe this is the first documented case in the literature of LS caused by *Streptococcus intermedius* occurring after a type B influenza infection.

## Case presentation

**Figure 1:**
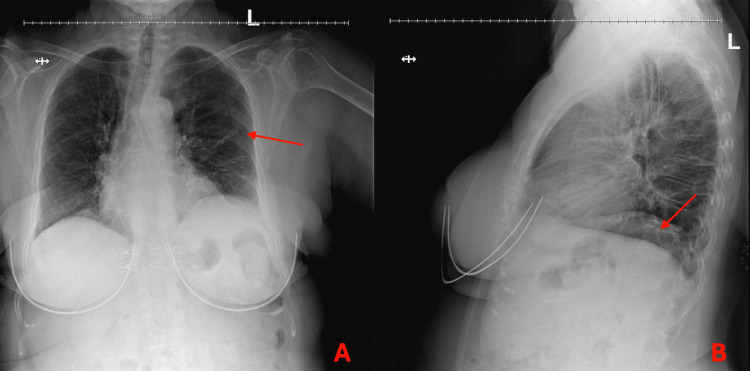
Chest X-ray A and B showing the anterior-posterior and lateral X-ray views of the chest, respectively. A shows left subsegmental atelectasis as indicated by the arrow. B shows a left lower lobe consolidation as indicated by the arrow

**Figure 2:**
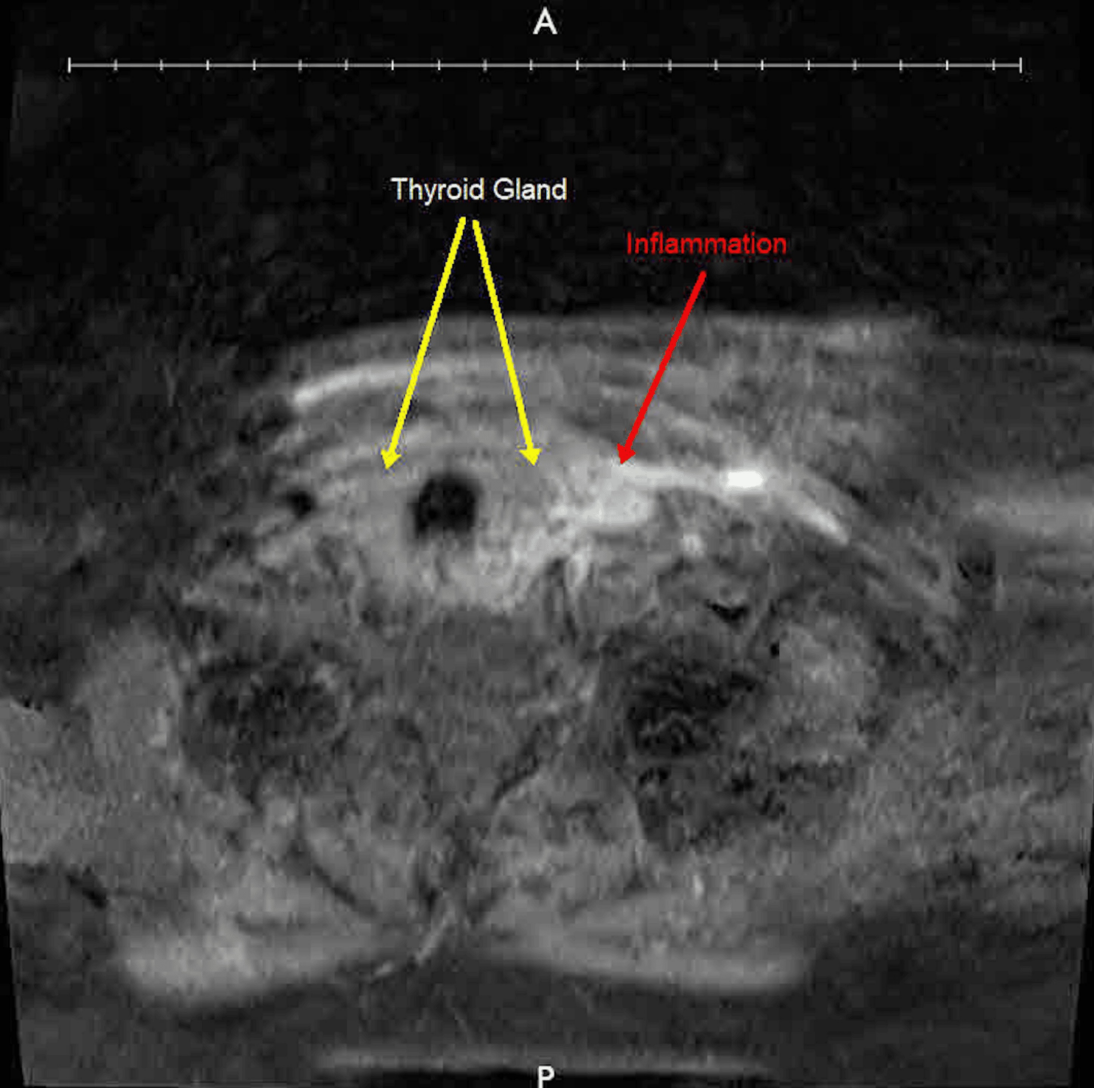
Magnetic resonance imaging (MRI) of the neck SCM: Sternocleidomastoid muscle Neck MRI without contrast showing an inflammatory reaction involving the left SCM and the neck soft tissues to the left of the thyroid gland (yellow arrows) with minimal fluid, but no drainable mature abscess, as indicated by the red arrow

An 89-year-old female with a history of hypertension presented to the emergency department (ED) with complaints of pain behind her left ear, radiating down her neck, and a sore throat for three days. The pain was worse in the morning and was aggravated by the movement of her head. She denied having a fever, chills, headache, visual changes, chest pain, cough, nasal congestion, or shortness of breath. The physical exam and vitals were significant for hypertension and showed tenderness upon palpation of the left occipital prominence and the left sternocleidomastoid muscle (SCM). Laboratory data (Table 1) revealed leukocytosis, hypokalemia, hypomagnesemia, hyperglycemia, elevated C-reactive protein, positive influenza type B antigen, and a negative rapid group A *Streptococcus *screen. Additionally, the peripheral blood culture was negative. Chest X-ray (Figure 1A-1B) showed left subsegmental atelectasis with a left lower lobe consolidation. Additionally, magnetic resonance imaging (MRI) of the neck with and without contrast (Figure 2) showed myositis of the left SCM without any obvious abscess. She was admitted for six days and received a five-day treatment of oseltamivir 30 mg twice daily and two days of intravenous (IV) cefepime 1 g every 12 hours and IV vancomycin 1250 mg every 24 hours. She was then discharged on a tapered steroid regimen, starting with 20 mg of prednisone daily.

**Table 1:** First admission laboratory results

First admission laboratory tests	Results	Reference range
Hemoglobin	14.6 g/dL	Male: 13.5-17.5 g/dL; female: 12.0-16.0 g/dL
Hematocrit	44.1%	Male: 41%-53%; female: 36%-46%
White blood cell (WBC)	21.37 K/uL high	4.5-11.0 K/uL
Platelet count	289 K/uL	150-00 K/uL
Sodium	141 mmol/L	135 - 145 mmol/L
Potassium	3.1 mmol/L low	3.70 to 5.20 mmol/L
Glucose	144 mg/dL high	70 - 100 mg/dL
Chloride	105 mmol/L	96 to 106 mmol/L
Magnesium level	1.7 mg/dL low	1.7 to 2.2 mg/dL
C-reactive protein (CRP)	304.2 mg/L high	<3 mg/L
Strep A screen	Negative ser	Negative
Pan-SARS Ag FIA	Negative	Negative
Influenza A antigen	Negative	Negative
Influenza B antigen	Positive, critical	Negative

Eight days after discharge, she returned to the ED for further evaluation due to three days of difficulty swallowing and worsening left-sided neck pain that intensified with movement. She denied having fever, chills, nausea, vomiting, or any history of dental infections. Examination of the neck revealed tenderness, swelling, and erythema with mild fluctuance over the anterolateral aspect of the left neck, specifically over the SCM muscle, accompanied by increased warmth. Vitals showed a significantly elevated blood pressure of 183/65. Mild coarse breath sounds were noted at the lung bases bilaterally; otherwise, the remainder of the physical exam was unremarkable. Laboratory tests at her second admission (Table 2) showed thrombocytosis, leukocytosis with a neutrophilic predominance, elevated sedimentation rate, elevated lactic acid, hyperglycemia, elevated blood urea nitrogen (BUN)/creatinine ratio, and elevated pro-B-type natriuretic peptide (Pro-BNP).

**Table 2:** Second admission laboratory results WBC: White blood cell; BUN: blood urea nitrogen; pro-BNP: pro-B-type natriuretic peptide

Second admission laboratory tests		Results	Reference ranges
Complete blood count (CBC)	Hemoglobin	12.1 g/dL	Male: 13.5-17.5 g/dL; female: 12.0-16.0 g/dL
	Hematocrit	37.8%	Male: 41%-53%; female: 36%-46%
	WBC	30.51 K/uL high	4.5-11.0 K/uL
	Platelet count	566 K/uL high	150-400 K/uL
Differential type	% Neutrophils	89.7% high	54%-62%
	% Immature granulocytes	1.1% high	0%-0.6%
	% Lymphocytes	3.5% low	25%-33%
Complete metabolic panel (CMP)	Sodium	137 mmol/L	135-145 mmol/L
	Potassium	4.4 mmol/L	3.70-5.20 mmol/L
	Glucose	183 mg/dL high	70-100 mg/dL
	Chloride	100 mmol/L	96-106 mmol/L
	BUN	25 mg/dL high	6-20 mg/dL
	Creatinine	0.93 mg/dL	0.6-1.3 mg/dL
	BUN/creatinine ratio	26.9 mg/dL high	10-20 mg/dL
	Magnesium level	2 mg/dL	1.7 to 2.2 mg/dL
	Sedimentation rate	31 mmol/hr high	0 to 15 mmol/hr
	C-reactive protein (CRP)	672 mg/L	<3 mg/L
	Lactic acid (lactate)	3 mmol/L high	<2 mmol/hr
	Pro-BNP	495 pg/mL high	<450 pg/mL if >75 years old

**Figure 3:**
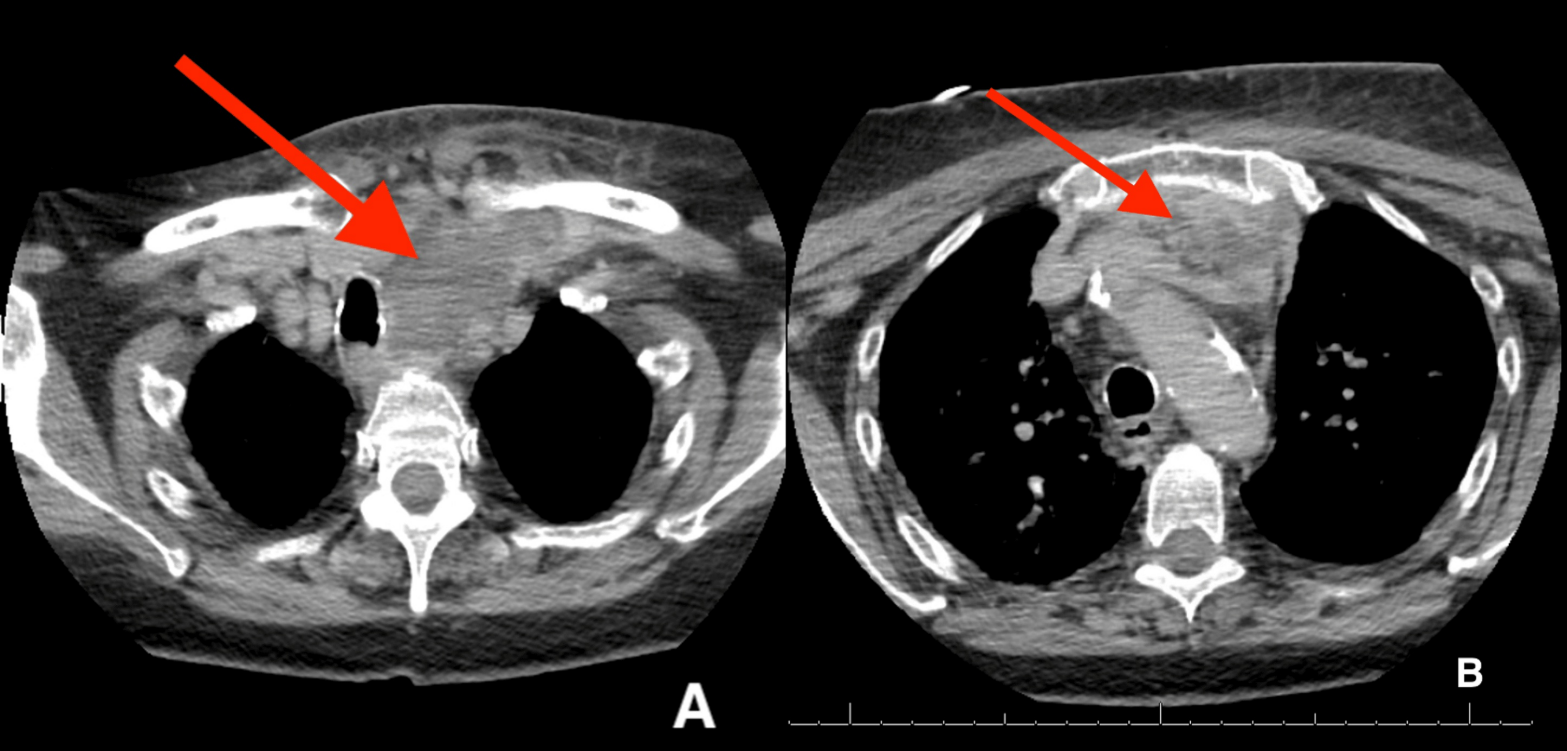
Neck CT with contrast CT: Computed tomography A and B showing two axial planes of the patient's neck CT with contrast. Arrows indicate a multiseptated collection in the left base of the neck, consistent with an abscess measuring approximately 7.0 x 4.5 x 5.4 cm. A shows the superior axial view of the abscess causing mass effect on the trachea 1.5 cm to the right of midline. B shows an inferior axial view of the abscess as it extends to the level of the aortic arch

A chest X-ray revealed increased interstitial opacities, more pronounced in the left lung than the right, indicative of pulmonary edema, although showing overall improvement from the chest X-ray taken on her previous admission. The electrocardiogram showed sinus rhythm with normal PR and QTC intervals, left axis deviation, and intraventricular conduction delay, all unchanged from her prior exams. Contrast-enhanced CT of the neck (Figure 3A-3B) exhibited marked progression from the mild inflammatory findings in the left neck of the prior MRI into an extensive, multiseptated peripherally enhancing collection in the left base of the neck, consistent with an abscess. The abscess extended into the superior mediastinum, measuring approximately 7.0 x 4.5 x 5.4 cm and causing mass effect on the trachea 1.5 cm to the right of the midline (Figure 3A). Additionally, thrombosis of the left internal jugular vein, and likely the left subclavian vein, was observed on the neck CT. Bilateral upper extremity venous duplex further confirmed the presence of an acute deep vein thrombosis in the left internal jugular vein.

While in the ED, she received a loading dose of IV piperacillin/tazobactam 4.5 g, IV dexamethasone 10 mg, IV morphine 2 mg, IV ketorolac 15 mg, and IV hydration with normal saline. The otolaryngology, vascular surgery, and infectious disease services were consulted. She was admitted to the intermediate medical care unit for further workup. Vascular surgery recommended anticoagulation therapy without endovascular intervention. Otolaryngology suggested a wide incision and drainage of the abscess due to suspicion for LS, although the underlying pathophysiology remained elusive at the time. Infectious disease recommended continuing treatment with piperacillin/tazobactam 3.375 g IV every eight hours and evaluating for methicillin-resistant *Staphylococcus aureus *via polymerase chain reaction, which was later confirmed to be negative. 

On the second day of admission, the patient was taken to the operating room for a wide incision and drainage of the left cervical/mediastinal abscess. Intraoperative findings revealed copious pus and necrotic tissue within the left neck at the level of the left thyroid gland extending inferiorly into the left upper mediastinum and diving retrosternally down to the level of the aorta. Three ½ inch Penrose drains were placed into the deep pockets of the incision to facilitate further drainage of the abscess and were removed on postop day seven.

Following the surgery, both peripheral blood cultures taken on admission returned positive for *Streptococcus intermedius* within 48 hours. At the recommendation of the infectious disease department, the patient was initially treated with IV piperacillin/tazobactam 3.375 g every eight hours for two days until being switched to IV ceftriaxone 2 g every 24 hours and IV metronidazole 500 mg every eight hours, as the blood culture sensitivity results indicated sensitivity to ceftriaxone and penicillin. At this point, the otolaryngologist made a final diagnosis of LS with a neck abscess.

The remainder of the patient’s hospital course was complicated by acute bacterial sialadenitis and diarrhea. The patient’s stool culture and *Clostridioides difficile* panel were negative for bacterial growth, showing only greatly reduced enteric flora. The diarrhea improved with the initiation of Florastor 250 mg twice daily, as well as as-needed loperamide 2 mg, and adjustment of IV metronidazole to every 12 hours. Additionally, the patient developed acute bacterial sialadenitis of the left parotid gland. The pain and discomfort in the left parotid gland subsided with supportive measures including warm compresses, “milking” of the gland, and continuation of her IV antibiotics.

Repeat blood cultures drawn 72 hours after admission were negative after 48 hours. By day eight of admission, the patient’s white blood cell and platelet levels had decreased to 11.11 K/uL and 434 K/uL, respectively. Both had returned to the normal range by day 10 of admission.

Upon discharge, the patient was advised to continue antibiotic treatment with oral amoxicillin/clavulanic acid 875 mg twice daily for a total of four weeks and to continue deep vein thrombosis prophylaxis with Eliquis (apixaban) 5 mg every 12 hours for approximately three to six months. She scheduled follow-up appointments with her primary care physician, otolaryngology, and hematology. After completing six months of anticoagulation treatment, she achieved a full recovery.

## Discussion

This case report demonstrates a unique and noteworthy presentation of LS due to an underlying *Streptococcus intermedius *infection following infection with influenza B. Most often LS is caused by *Fusobacterium necrophorum*, and there are few cases reported of this condition occurring due to *Streptococcus intermedius* [5]. A distinguishing feature of this case is that the patient’s condition likely originated due to an initial influenza B infection and SCM myositis, followed by a *Streptococcus intermedius-*induced abscess formation, which led to the subsequent development of LS. 

This case is additionally unique in that this syndrome is rarely seen in patients of this age. LS primarily affects individuals aged 10 to 35 years. Nonetheless, it is critical to recognize that LS can manifest in patients of all age groups. The case of our 89-year-old patient highlights the importance of considering this syndrome as a differential diagnosis, even in older individuals who fall outside the typical age range for LS. This patient had a recent viral infection that preceded her bacterial infection. However, we do not know if she may have had a condition or additional risk factors that predisposed her to a hyperviscous and prothrombotic state. An additional limitation of this case report is that our patient had a single comorbid condition of hypertension and that may not be generalizable to the population with multiple comorbidities in this age group. 

Although the patient's two presentations to the ED did not follow the usual clinical presentation of LS, her case highlights the need for physicians to thoroughly evaluate patients with unexplained, worsening neck pain for this rare disease. The patient's hospital course included obtaining an MRI as one of the original imaging modalities to evaluate her neck pain. This scan was initially unremarkable for an abscess formation, and only demonstrated myositis of the SCM. However, repeat neck CT, which is the more commonly used imaging study in LS diagnosis, showed signs of an abscess. After the blood cultures came back positive and the definitive diagnosis was made, antibiotic treatment with IV piperacillin/tazobactam followed by IV ceftriaxone and metronidazole was utilized. This led to clearance of the bacterial infection after 48 hours, as demonstrated by the negative repeat peripheral blood cultures. This is in line with the recommended use of carbapenem or piperacillin/tazobactam in combination with metronidazole. 

As more cases of LS are reported, it is evident that this unusual condition can present in diverse ways. This case demonstrates the importance of thoroughly investigating a patient's symptoms, rapidly assessing their condition, and promptly draining the abscess to achieve a successful clinical outcome. Hopefully, ongoing recognition of the varied clinical features of LS, along with research into its pathophysiology, will continue to enhance our understanding of this syndrome.

## Conclusions

We believe this to be the first reported case in the literature of LS secondary to *Streptococcus intermedius *presenting after infection with type B influenza. The patient's clinical course and the successful management of her disease offers valuable insights for treating similar cases, further contributing to our understanding and management of this rare syndrome. As more atypical LS cases emerge, it becomes increasingly clear that this condition can manifest in diverse ways, highlighting the importance of considering LS in a wider age range of patients presenting with neck pain and *Streptococcus intermedius *infection.

## References

[1] Allen BW, Anjum F, Bentley TP (2023). Lemierre Syndrome. StatPearls [Internet].

[2] Veras RO, Barasuol LL, de Lira CP, Klostermann FC, Müller LS, Nercolini LE, Nogueira GF (2018). Lemierre syndrome: case report. J Vasc Bras.

[3] Jafri FN, Shulman J, Gómez-Márquez JC, Lazarus M, Ginsburg DM (2018). Sore throat, fever, septic emboli, and acute respiratory distress syndrome: a case of Lemierre syndrome. Case Rep Emerg Med.

[4] Golpe R, Marín B, Alonso M (1999). Lemierre's syndrome (necrobacillosis). Postgrad Med J.

[5] B K A, Gilotra T, Tymko C, Siddique Z, Eranki A (2020). A rare case of Lemierre's syndrome caused by Streptococcus intermedius, presenting as an epidural abscess. Cureus.

[6] Gupta S, Merchant SS (2012). Lemierre's syndrome: rare, but life threatening-a case report with Streptococcus intermedius. Case Rep Med.

[7] Jacobs JA, Pietersen HG, Stobberingh EE, Soeters PB (1995). Streptococcus anginosus, Streptococcus constellatus and Streptococcus intermedius: clinical relevance, hemolytic and serologic characteristics. Am J Clin Pathol.

[8] Issa E, Salloum T, Tokajian S (2020). From normal flora to brain abscesses: a review of streptococcus intermedius. Front Microbiol.

[9] Yanagi H, Ozawa H (2020). Lemierre's syndrome complicating influenza A virus infection. J Gen Fam Med.

[10] Johannesen KM, Bodtger U (2016). Lemierre's syndrome: current perspectives on diagnosis and management. Infect Drug Resist.

[11] Salami A, Assouan C, Garba I, Konan E (2019). An unusual cause of Lemierre syndrome. J Stomatol Oral Maxillofac Surg.

[12] Campo F, Fusconi M, Ciotti M, Diso D, Greco A, Cattaneo CG, de Vincentiis M (2019). Antibiotic and anticoagulation therapy in Lemierre's syndrome: case report and review. J Chemother.

[13] Walkty A, Embil J (2019). Lemierre's syndrome. N Engl J Med.

[14] Bonny V, Hourmant Y, Mirouse A, Valade S (2019). A prostatic Lemierre syndrome. Int J Infect Dis.

